# Ribosome display for the rapid generation of high-affinity Zika-neutralizing single-chain antibodies

**DOI:** 10.1371/journal.pone.0205743

**Published:** 2018-11-16

**Authors:** Adinarayana Kunamneni, Chunyan Ye, Steven B. Bradfute, Ravi Durvasula

**Affiliations:** 1 Center for Global Health, Department of Internal Medicine, University of New Mexico, Albuquerque, New Mexico, United States of America; 2 Department of Medicine, Loyola University Medical Center, Chicago, United States of America; US Naval Research Laboratory, UNITED STATES

## Abstract

**Background:**

Zika virus (ZIKV) is an emerging pathogen with no approved therapeutics and only limited diagnostics available. To address this gap, six mouse single-chain antibodies (scFvs) to ZIKV envelope (E) protein were isolated rapidly and efficiently from a ribosome-displayed antibody library constructed from the spleens of five immunized mice.

**Methodology/Results:**

In this report, we have generated a panel of mouse scFvs to ZIKV E protein using ribosome display. The six scFvs demonstrated no cross-reactivity with DENV2 NGC envelope protein, suggesting specificity for ZIKV E protein. These scFvs showed differences in their affinity: two (scFv45-3, scFv63-1) of them were dominant after four rounds of panning, and showed higher affinity (an apparent Kd values from 19 to 27 nM) than the other four (scFv5-1, scFv7-2, scFv38-1, and scFv51-2). All six scFvs showed ZIKV-neutralizing activity in the plaque reduction neutralization test (PRNT) assay and their neutralizing activity was positively correlated with their affinities.

**Conclusions/Significance:**

The scFvs (45–3 and 63–1) with highest affinity may have dual utility as diagnostics capable of recognizing ZIKV E subtypes and may be further developed to treat ZIKV infection. Our approach has the added advantage of generating Fc receptor-deficient antibodies, minimizing concern of antibody-dependent enhancement (ADE) of infection.

## Introduction

The recent ZIKV outbreak is a health crisis with global repercussions. Rapid spread of the disease within the epidemic regions coupled with migration of infected persons has underscored the need for rapid, robust and inexpensive diagnostic tools and therapeutics. There are very few readily available monoclonal antibodies for ZIKV, severely limiting development of antibody-based diagnostics [1]. Experimental antibody-based therapeutics in animal models have shown that some antibody preparations are protective, but others enhance ZIKV infection [2–4]. This is due to the phenomenon of antibody-dependent enhancement (ADE), in which non-neutralizing antibodies enhance viral infection via interaction of the antibody constant region with cellular Fc receptors. Additional concerns exist regarding potential ZIKV mutants, which may not be detected by or treated with specific monoclonal antibodies. Currently, there is no approved vaccine or therapeutic for ZIKV infection. Therefore, it is of great interest to develop neutralizing anti-ZIKV single -chain antibodies (scFvs) for potential diagnostic and therapeutic purposes.

Ribosome display of antibody libraries has provided a powerful tool for the selection of mAbs and scFvs to important viral pathogens [5–7]. This display technology allows for rapid *in vitro* selection and evolution of antigen-specific antibodies from large antibody libraries in a cell-free system [8–11] and high affinity antibodies with desired specificity can be isolated by panning on the antigens of interest [6, 12, 13]. Because of small size, homogeneity and ease of genetic manipulation, scFvs offer significant benefits over traditional monoclonal antibodies, such as speed of development, consistency, and ease of optimization [14–17]. Additionally, concerns over ADE are eliminated with scFvs, since they lack the Fc region that is required for enhanced disease. However, their utility may be limited by the presence of HAVH autoantibodies in many patients [18].

In this study, we generated six unique neutralizing scFv antibodies against the ZIKV E protein from the spleens of immunized mice by antibody ribosome display technology. The resulting antibodies have been characterized in a range of *in vitro* assays which demonstrate diagnostic and therapeutic potential.

## Materials and methods

### Ethics statement

Mice experimental procedures were approved by the Institutional Animal Care and Use Committee (IACUC) at the University of New Mexico. Animal research performed at the University of New Mexico was conducted under an IACUC approved animal protocol in compliance with the Animal Welfare Act and other federal statutes and regulations relating to animals and experiments involving animals and adheres to principles stated in the *Guide for the Care and Use of Laboratory Animals* [19]. The University of New Mexico is fully accredited by the Association for the Assessment and Accreditation of Laboratory Animal Care International.

### Plasmids, strains and reagents

All reagents used in the study were commercially available and were of reagent grade or better. All restriction enzymes and DNA modification enzymes were of molecular biology grade. All primers were purchased from Invitrogen and Integrated DNA Technologies (IDT). pGEM-T easy cloning vector and TNT T7 Quick for PCR DNA kit (rabbit reticulocyte cell free extract) were purchased from Promega. Rosetta-gami (DE3) Competent Cells and pET32a Plasmid were purchased from Novagen (USA). Rabbit anti-Strep tag II polyclonal antibody affinity purified HRP conjugated, mouse anti-his antibody and goat anti-mouse HRP antibody were purchased from GenScript Inc. Dengue virus serotype 2 envelope [DENV2 New Guinea C (NGC)] protein was kindly supplied by Dr. Shelton S. Bradrick (University of Texas Medical Branch, USA). Anti-Dengue Virus Type II Antibody (clone 3H5-1) was purchased from MilliporeSigma (USA). Anti-ZIKV E antibody and purified ZIKV E protein were purchased from Alpha Diagnostic International (USA). ZIKV PRVABC59 was obtained from Dr. Robert Baker, Division of Microbiology and Molecular Biology, Illinois Institute of Technology Research Institute (Chicago, IL).

### Immunization of mice

BALB/c mice were obtained from The Jackson Laboratory. Mice (n = 5) were infected with ZIKV PRVABC59, and infection was confirmed with a positive ELISA. Splenocytes of individual vaccinated mice were harvested 6 wk following infection and were placed in 10 ml of TRIzol for use in RNA isolation.

### Rapid generation of single chain antibodies by ribosome display

#### Antibody library construction

Total spleen RNA was prepared as described by Azizi *et al*. [20]. Mice spleens were minced and homogenised in 10 ml TRIzol (Invitrogen). The total RNA pellet was air-dried and resuspended in 500 μl of nuclease-free water (stored at -80°C). Complementary DNA (cDNA) was synthesized from approximately 25 μg of total RNA using a SuperScript II RT (Invitrogen) following the manufacture’s instructions provided.

For antibody library construction, the PCR primers were based on published sequences [20] with minor modifications (see S1 Table). The primers were designed to introduce in-frame NcoI and NotI restriction sites to the 5’ end of the heavy-chain (VH) sequence and to the 3’ end of the light-chain (VL) sequence, respectively. The VH_F/VH_R and VL_F/VL_R sets of primers (see S1 Table) were used for PCR amplification of VH and VL gene segments using the cDNA template. The VH_R and VL_F set of primers (see S1 Table) were used to introduce overlapping sequences which enabled the scFv gene fragments to be assembled by overlap extension PCR and these primers encode a 20 amino acid linker sequence (G_4_S)_4_. Amplified VH and VL PCR products were gel purified and pooled. An aliquot of VH and VL templates was subjected to overlap extension PCR amplification using Link to introduce Strep II tag and Kozak sequence on the 5’ end and an overlap extension on the 3’ end to facilitate joining to the variable heavy-chain libraries using MVKR and KzSTREPII. Finally, the PCR product encoding all the variable heavy-chain and light-chain combinations was amplified with primers RDT7 and MVKR to introduce T7 site into Strep tag II-conjugated VH-VL library and to produce the DNA encoding the anti-ZIKV immunoglobulin scFv libraries. The initial PCR amplification reactions were performed at a 52°C annealing temperature with 30 cycles, and the subsequent library assembly step used 16 cycles with *GoTaq* DNA polymerase and 20 pmol of each primer pair per reaction. DNA fragments were resolved by gel electrophoresis on 1% (wt/vol) agarose gels. DNA isolation from agarose gels was carried out following QIAquick DNA gel purification kit instructions. The final purified PCR product ~0.8 kb is a template for Ribosome Display.

#### Cell-free ribosome display technology

To select specific antibody fragments, we have used a modified eukaryotic ribosome display with slight modifications [21]. *In vitro* transcription and translation reaction was based on a coupled rabbit reticulocyte lysate system (Promega’s TNT quick-coupled transcription-translation system) and performed according to the supplier’s protocol. The PCR-generated DNA library of antibody-coding genes derived from the spleens of five mice were expressed in this lysate system. Briefly, 50 μl of transcription/translation mixture containing 40 μl of TNT T7 Quick Master Mix, 2 μl of DNA library (0.1 to 1.0 μg), 1 μl (1 mM) of methionine, 1 μl of DNA enhancer, and 6 μl of water were added, and the reaction mixture was incubated at 30°C for 90 mins. Then, 5 μl of RNase-free DNase I (Roche) (2,000 U/ml) was added, and the mixture was incubated for 20 mins at 30°C (in order to remove the DNA template so that subsequent PCR only picks up pulled down RNA sequences).

To select specific antibody fragments the 0.5 ml PCR tubes were coated with 1 μg/ml of the recombinant ZIKV E protein in 100 μl PBS at 4° C overnight. Protein coated tubes were washed with PBS and blocked with 100 μl of molecular biology grade bovine serum albumin (BSA) in PBS (10 mg/ml) (New England Biolabs) for 1 hour at room temperature. The translation/transcription mixture [containing the protein-ribosome-mRNA (PRM) complexes] was added to the washed and blocked protein-coated tubes and incubated on ice for 1 hour. The PCR tubes were washed three times with ribosome display washing buffer (PBS containing 0.01% Tween 20, 5 mM Mg acetate and 0.1% BSA, pH 7.4) and two times quick wash with ice-cold RNase-free water, and the retained RNA (antibody sequences) subjected to the following recovery process; *in situ* Single-Primer RT-PCR Recovery was performed in the PCR tubes carrying selected ARM complexes using a SuperScript II reverse transcriptase (Invitrogen, USA). The obtained cDNA was amplified in a 25 μl PCR mixture for 35 cycles of 30 s at 94°C, 30 s at 65°C, and 1 min at 72°C, and 10 mins at 72°C with MKR and KzStrep II using *GoTaq* DNA polymerase (Promega, USA). The RT-PCR product from a single round of ribosome display was purified by agarose gel electrophoresis. The purified PCR products were used for the next round of ribosome display.

#### Cloning, expression and purification of an anti-ZIKV E scFvs

The RT-PCR product from a fourth round of ribosome display was cloned into the pGEM-T Easy vector (T-Cloning Kit, Promega) according to the manufacturer’s instructions. The ligation products were transformed in pGEM-T Easy vector *E*. *coli* cells (XL1-Blue) and positive colonies (~100) were chosen by blue-white selection and confirmed by DNA sequencing using T7 and SP6 standard primers (S1 Table). Based on the sequencing results, the clones in right reading frame without stop codon were chosen for prokaryotic expression.

For the construct of scFvs, DNA was amplified with the forward primer, KzStrepII 5’CGAATTCCA**CCATGG**CCTGGAGCCATCCGCAGTTCGAGAAGACCGGCAGCGG3’ (with an NcoI restriction sequence highlighted in bold) and the reverse primer, MVKR 5’AGT**GCGGCCGC**ATCAGCCCGTTTTATTTCCAA3’ (with an Not I restriction sequence highlighted in bold). Both set of primers allow these two amplicons to be subcloned into a pET32a-His vector and were transformed into Rosetta Gami (DE3) *E*. *coli* strain and plated onto LB agar plate with 100 μg/ml of carbenicillin, and grown at 37°C for 16 hours. The molecular weight and isoelectric points were predicted using ExPASy bioinformatics resource portal (http://web.expasy.org/compute_pi/) [22]. Next day, five colonies were inoculated in 3 ml of LB media with the same antibiotics and grown at 37°C with 225 rpm shaking for 16–18 hours and the positive clones were selected by restriction analysis with NcoI and NotI and confirmed by DNA sequencing. The 3 ml overnight culture was used to prepare a glycerol bacterial stock and inoculated into 200 ml of LB medium containing the same antibiotics and grown at 37°C with shaking at 225 rpm until OD_600_ reached between 0.4–0.6. The culture was briefly chilled on ice to 25°C and the cells were induced by the addition of IPTG (final concentration 1 mM) and were incubated for 12 hours at 25°C with shaking. Cells were harvested in 4x50 ml tubes by centrifugation at 4000 *g*, 4°C for 20 mins. Cell pellets were frozen and stored at -80°C before undergoing further processing.

The 50 ml cell pellet from 200 ml culture was re-suspended in 3 ml of lysis buffer (20 mM Tris–HCl, 500 mM NaCl, 20 mM Imidazole, 0.1% Triton X-100 pH 8.0). Cells were lysed by sonication on ice (6x30 seconds), and were centrifuged at 14,000 *g* for 15 mins to remove cellular debris. The soluble fraction was filtered through 0.2μm filters and applied to HisTrap excel 1ml column (GE Life Sciences, USA). The column was equilibrated and washed with 20 mM Tris–HCl, 500 mM NaCl, 20 mM Imidazole pH 8.0 and the sample was eluted 20 mM Tris–HCl, 500 mM NaCl, 500 mM Imidazole, pH 8.0 in one elution step. The purification was carried out at a constant flow of 1 ml/min. Fractions of 1 ml were collected through the elution step. The purified protein fractions were kept at 4°C.

#### Western blot of scFv fragments

The proteins were separated by SDS-PAGE and were blotted onto polyvinylidene difluoride (PVDF) membrane using an iBlot2 Gel TransferDevice (Life Technologies) for 7 mins. The membranes were blocked in 5% w/v skimmed milk powder/TBS buffer at room temperature for 1 hour, then incubated with mouse anti-His antibody (GenScript, Piscataway, NJ, USA) at a dilution of 1:5000 for 1 hour. After three washes with TBST buffer, HRP-conjugated Donkey anti-mouse antibody (diluted 3/10,000 in TBST) (Abcam, USA) at room temperature for 1 hour, followed by washing with TBS-T buffer 3x for 10 mins each. The protein was detected and visualized with Amersham ECL detection reagent (GE Healthcare, USA) following the manufacturer’s recommendation.

#### Analysis of purified scFvs

Binding specificity and cross-reactivity of anti-ZIKV scFv antibodies was determined in ELISAs. Nunc Polysorp ELISA plates were coated with target ZIKV E or DENV2 envelope protein antigens (200 ng/well) overnight at 4°C, blocked with 2% BSA at 37°C for 1 hour and incubated with anti-ZIKV scFvs in 1% BSA. Anti-ZIKV E antibody (Alpha Diagnostic Intl Inc., USA) and anti-Dengue Virus Type II Antibody were used against ZIKV E and DENV2 (NGC) E proteins as positive controls, respectively. Mouse anti-EBOV scFv4-2 (unpublished data) was used as negative control. After 1 hour incubation at 37°C, plates were washed five times and scFv was detected with 3:10 000 dilution of HRP-conjugated Rabbit anti-strep tag II polyclonal antibody (Genscript, USA) in PBS containing 1% BSA and incubated at 37°C for 30 mins. After washing and tap drying, 100 μl of TMB substrate was added to each well and the plate was allowed to incubate for 15–30 mins at 25°C. The reaction was stopped with 0.18M H_2_SO_4_, and absorbance reading was measured at 450nm and these values were transformed using GraphPad Prism 5 software.

To determine binding affinity (an apparent Kd), scFvs were tested for binding to the protein ZIKV E (200 ng/well) at a concentration range of 200 nM to 0.01 nM. ELISAs were performed as described above.

#### ZIKV plaque reduction neutralization test (PRNT)

Vero-E6 cells were seeded onto 12-well plates and incubated at 37°C for 12–24 hours to 90% confluency. A 50–100 pfu/200 ul dilution of ZIKV (Asian genotype, strain PRVABC59) in media in sterile Eppendorf tubes was made in media and mixed with equal volumes of 2-fold dilutions of scFvs. This mixture was incubated at 37°C for 1–1.5 hours, added to cells, and incubated at 37°C for 2 hours. Media was aspirated from the wells, and cells washed once with PBS. 1 ml/well of viral overlay media (equal volumes of 2% Agarose and 2 X Modified Eagle’s Media with serum) was overlayed on cells, and cells were incubated at 37°C for 5–7 days. The cells were then fixed with 4% formaldehyde for at least 2 hours. After aspiration, the plate was turned upside down and the agarose overlay gently teased loose with a metal spatula and discarded without damaging the cell monolayer. The monolayer was gently washed with water to remove residual agarose. 0.5% Crystal violet was added to well for 1–2 min, the wells dried, and plaques counted manually. The percentage inhibition of virus infectivity is plotted as a function of scFv concentration and PRNT_50_ values for the inhibition curves are calculated by using GraphPad PRISM 5 Software.

## Results

### Anti-ZIKV scFv antibodies generated from cell-free ribosomal display

**Fig 1:**
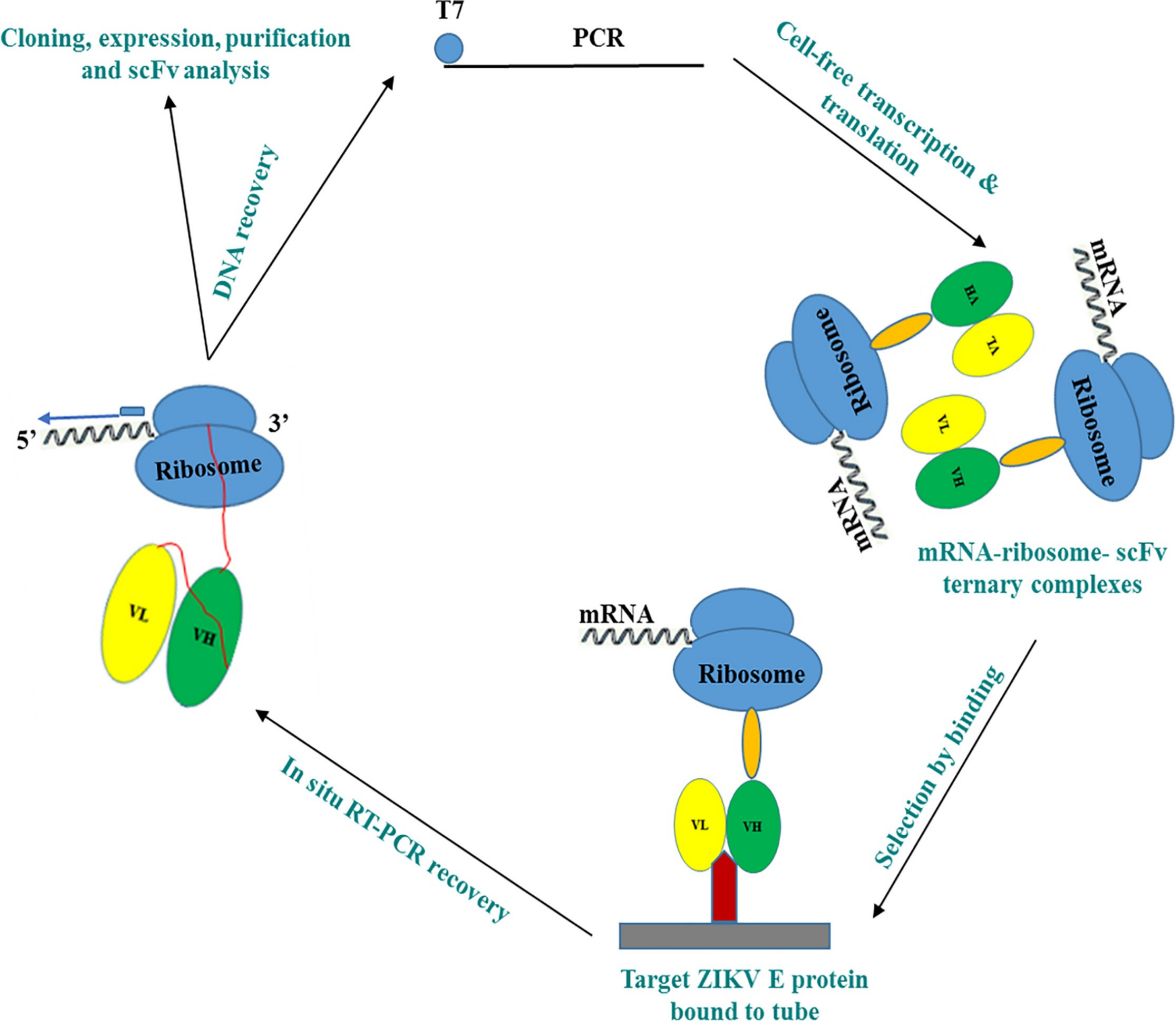
Graphical overview: scFV’s were generated rapidly using cell-free ribosomal display from mice immunized with flavivirus ZIKV PRVABC59.

**Fig 2:**
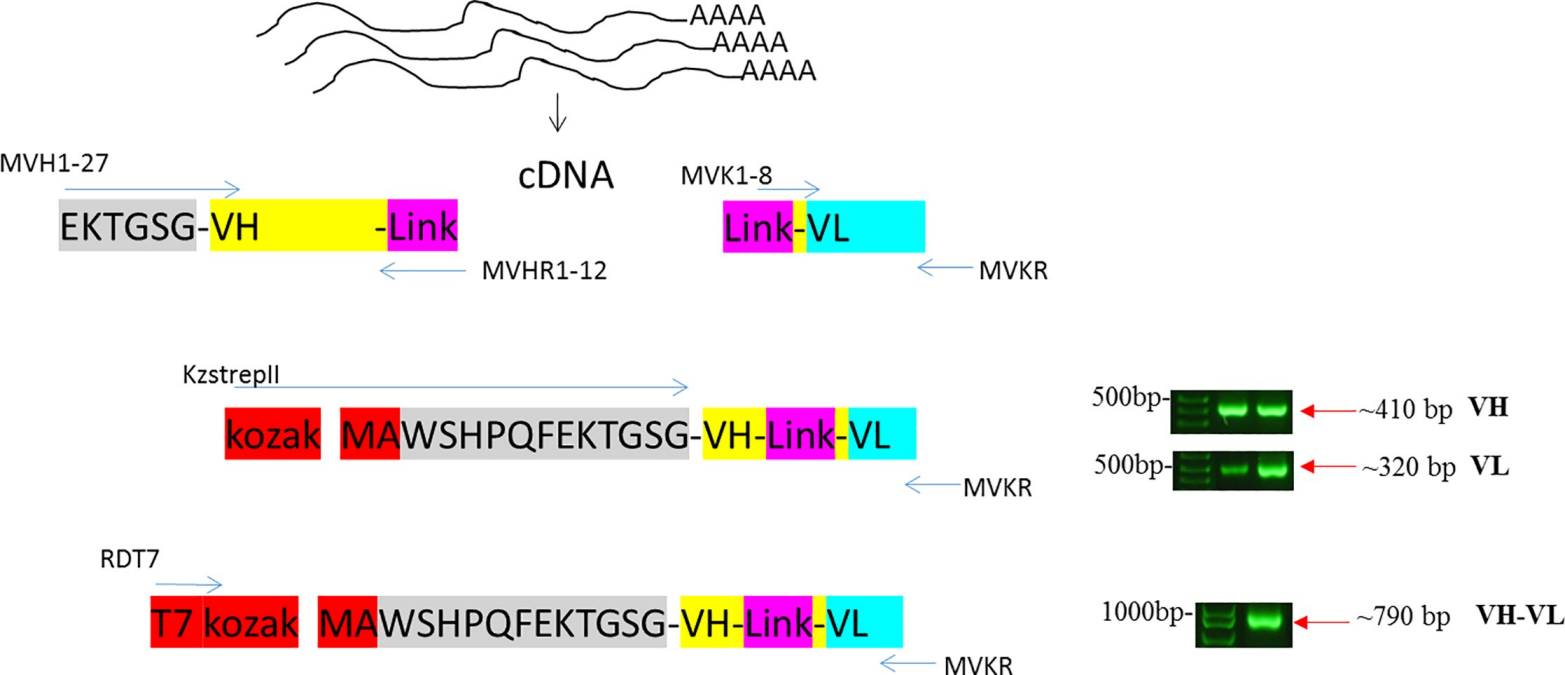
An illustration of the mouse immunoglobulin library assembly process showing the PCR amplification and assembly steps using mouse spleen total RNA.

**Fig 3:**
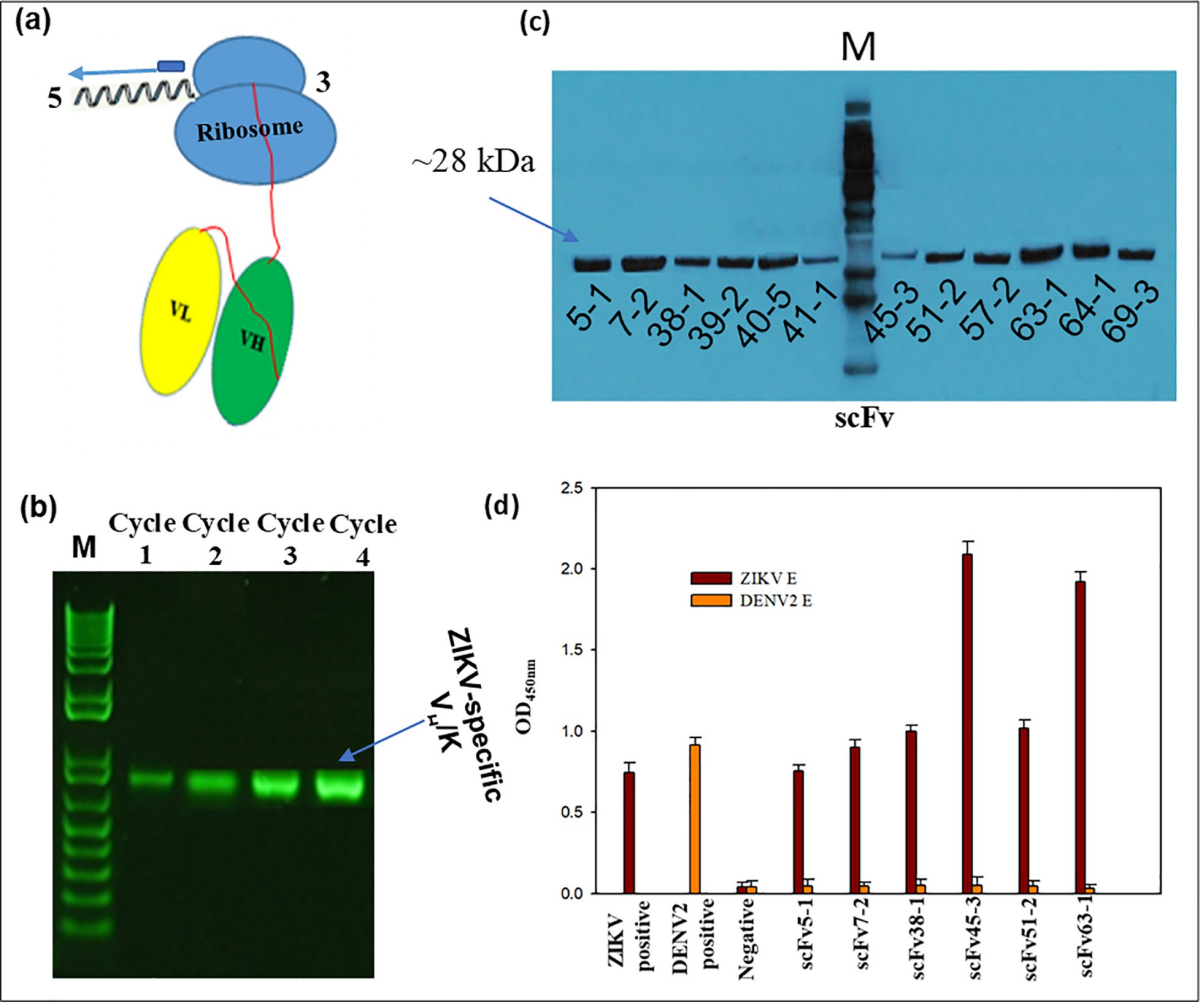
(a) Schematic of stalled ARM complex and position of primers used for RT-PCR recovery in the 1^st^, 2^nd^, 3^rd^ and 4^th^ cycles of ribosome display. T7 is the 5’ primer and MVKR is the 3’ primer. (b) Analysis of RT-PCR recovery of V_H_/K cDNA from ZIKV-immunized spleen in the 1^st^, 2^nd^ , 3^rd^ and 4^th^ cycles. V_H_/K complexes were bound to ZIKV E protein coated to PCR tube through 4^th^ cycle of selection and recovery. (c) Western blot of purified 12 unique anti-ZIKV scFvs generated by ribosomal display. (d) Binding specificity and cross-reactivity of anti-ZIKV E scFvs. ELISA graph showing ZIKV positive control (anti-ZIKV ENV antibody), DENV2 positive control (Anti-Dengue Virus Type II Antibody), negative control (Mouse anti-EBOVscFv4-2) and 6 scFv antibodies (5–1, 7–2, 38–1, 45–3, 51–2, and 63–1) against ZIKV E and DENV2 E proteins. Bound antibodies were detected by HRP-conjugated rabbit anti-strep tag mouse IgG antibody. Each point represents the mean values of triplicate wells and the standard deviation is represented by error bars.

For ribosome displayed scFv antibody libraries, the cDNAs encoding the immunoglobulin VH and VL regions joined by a 20 amino acid flexible linker [(G_4_S)_4_] were constructed using total RNA isolated from the spleens of five mice immunized with ZIKV PRVABC59 (Figs 1 and 2, S1 Table). The ribosome-displayed scFv library was panned against ZIKV E protein with 4 rounds of selection. PCR products were cloned into pGEM-T vector and the V_H_—V_L_ transformants (50 clones) were randomly selected for sequencing, resulting in the isolation of a panel of 12 anti-ZIKV recombinant antibodies that were expressed and purified from *E*. *coli* cytoplasm (Fig 3); sequencing verified that all 12 antibodies were novel (S1 Fig, S2 Table). Genes were subcloned into a pET32a expression vector, expressed, and purified from *E*. *coli* Rosetta**-**gami cytoplasm. Yields of 3 to 5 mg/l of pure, soluble, active scFv fragments were obtained from shake flask cultures.

We have identified six promising scFv clones (5–1, 7–2, 38–1, 45–3, 51–2 and 63–1) by indirect ELISA; all other scFv antibodies were negative (data not shown). To determine whether the recombinant scFvs could bind specifically to their corresponding ZIKV E protein, we examined the six soluble, purified scFv antibodies by ELISA assays (Fig 3D). They reacted only with the specific ZIKV E protein that was used to screen the library and did not cross-react with the flavivirus DENV2 virus NGC strain E protein (Fig 3D). scFvs 45–3 and 63–1 showed the highest reactivity while scFv 5–1 showed the lowest reactivity. The reactivities of 7–2, 38–1 and 51–2 ranked lower than those of 45–3 and 63–1, in that order.

Sequence analysis indicated that many unique specific scFv clones were isolated by ribosome display selections (S1 Fig and S2 Table). The heavy chain and light chain gene families of the isolated clones (5–1, 7–2, 38–1, 45–3, 51–2 and 63–1) were designated based on VBASE2 Ig database (http://www.dnaplot.com), indicating all the sequenced clones are mouse immunoglobulin genes and germline-derived (S2 Table). The heavy chains of scFv 38–1, 45–3 and 63–1 belong to the VJ558 VH1 gene family and that of 5–1 and 51–2 belongs to the VSM7 VH14 gene family, whereas 7–2 belongs to the VSM7 VH14 gene family. The light chain of scFv7-2 and 63–1 belongs to IGKV2 subgroup and those of 5–1, 38–1, 45–3 and 51–2 belong to IGKV1, IGKV12/13, IGKV4/5, and IGKV21 subgroups, respectively. scFv5-1 and scFv51-2 have the same VH sequences (heavy chain), but they have different VL sequences. As expected, most of the clones represented different germline VH segments and also had somatic hypermutations; these clones had distinct combinations of heavy and light chains. The results of the sequence analyses are summarized in S2 Table.

### ZIKV plaque reduction neutralization test (PRNT) to measure neutralizing scFv antibody activity

**Fig 4:**
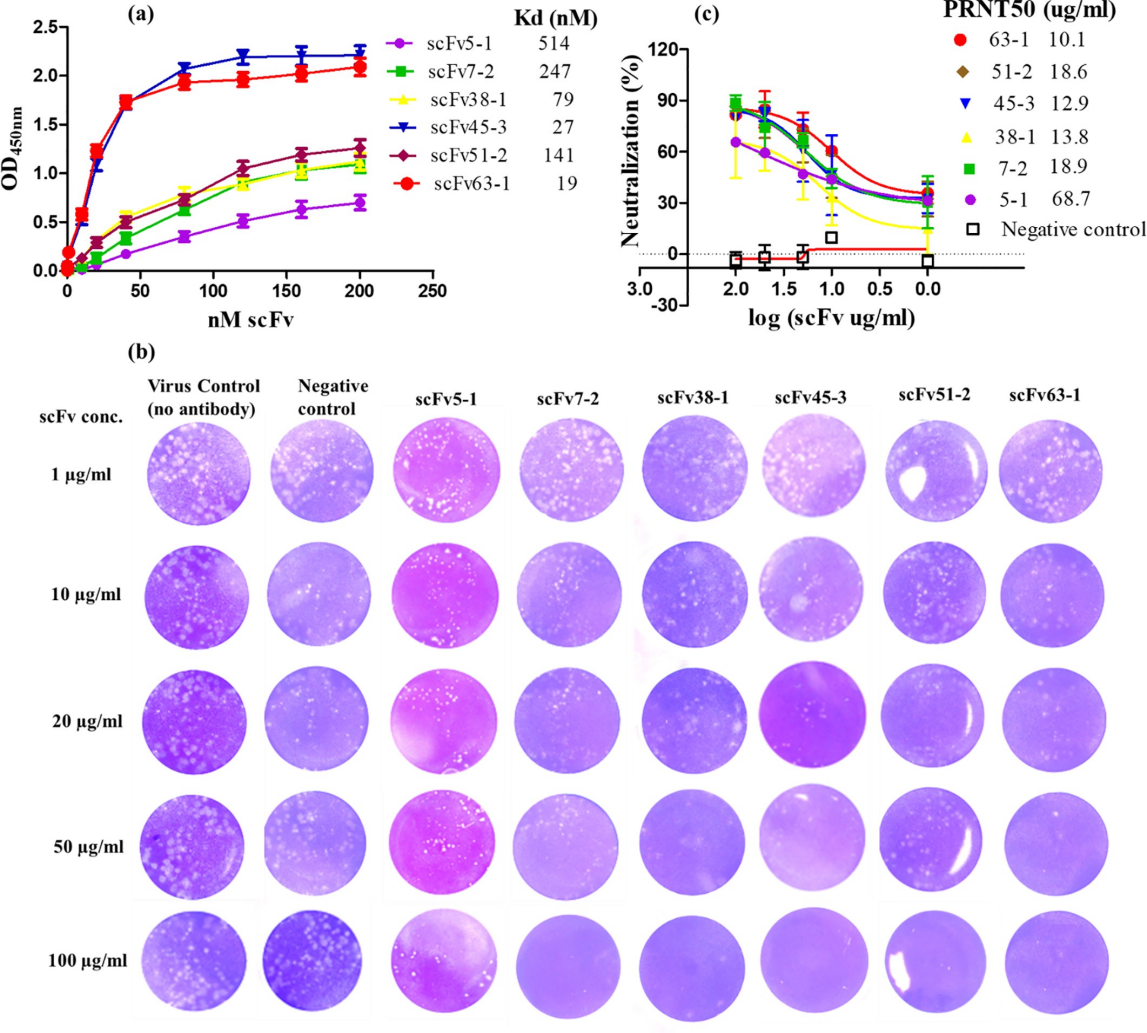
(a) ZIKV-binding affinity[1] of purified scFvs, 5–1, 7–2, 38–1, 45–3, 51–2 and 63–1. Dilutions of the purified antibodies were added to microtiter wells coated with ZIKV (200 ng/well). Bound antibodies were detected by HRP-conjugated rabbit anti-strep tag mouse IgG antibody. Each point represents the mean ±the s.d. values of triplicate wells. (b) Plaque reduction (PRNT) assay for ZIKVPRVABC59 neutralization by anti-ZIKV scFvs. The “virus control” represents the result obtained by infection of virus only without addition of antibody. ZIKV was tested against varying concentrations (1, 10, 20, 50 and 100 μg/ml) of each scFv antibody. A murine scFv4-2 is an irrelevant antibody (EBOV-specific) used as negative control. (c) Neutralization dose–response curve- the PRNT data for each antibody at each point were used to calculate % reduction in plaques, determined as follows: % neutralization = [(plaque in the absence of antibody–plaque in the presence of antibody) / plaque in the absence of antibody] × 100. The PRNT50 values were measured to predict the therapeutic potency of scFvs by evaluating cytopathic effects in infected Vero cells. The results shown are the average±the s.d. of three replicates.

The six scFvs were characterized for ZIKV neutralization by a plaque reduction neutralization test (PRNT). Three hundred microliters of ZIKV (Asian genotype (PRVABC59) were incubated with an equal volume of media containing each scFv at varying concentrations at room temperature for 1 hour. After addition of the ZIKV/scFv mix, cells were cultivated for 5–7 days, and plaques were visualized and counted (S3 Table). Fig 4 shows the results of PRNT using each scFv. All six scFvs showed neutralizing activity against ZIKV, whereas an irrelevant control scFV antibody did not block ZIKV infection. scFvs 45–3 and 63–1 completely inhibited the ZIKV infection at a concentration of 50 μg/ml and reduced the number of plaques by 80% at a concentration of 20 μg/ml whereas 7–2, 38–1 and 51–2 completely blocked the ZIKV infection at a concentration of 100 μg/ml and reduced the number of plaques by 80% at a concentration of 50 μg/ml. scFv5-1 did not inhibit completely ZIKV infection at a concentration of 100 μg/ml. The scFvs were thus able to potently inhibit ZIKV infection, with 50% inhibition occurring at titers of 68.7 μg/ml (for scFv5-1), 20.1 μg/ml (for scFv7-2), 14.5 μg/ml (for scFv38-1), 12.9 μg/ml (for scFv45-3), 17.0 μg/ml (for scFv51-2) and 10.1 μg/ml (for scFv63-1) (Fig 4B and 4C). The neutralizing activities of the six scFvs were positively correlated with their affinities to ZIKV (Fig 4A–4C).

## Discussion

A diverse set of mouse scFvs can be obtained rapidly and efficiently from a ribosome antibody library generated from immunized mice. In this study, we isolated six different ZIKV neutralizing mouse scFvs (5–1, 7–2, 38–1, 45–3, 51–2, and 63–1) by ribosome display of the antibody library generated from the spleens of five mice immunized with ZIKV. The scFvs 5–1, 7–2, 63–1, 38–1 and 45–3 have highly homologous VL sequences, whereas 51–2 has a ~35% different VL sequence (S1 Fig**)**. Importantly, these scFvs have markedly divergent VH sequences including CDRs (S1 Fig and S2 Table), and also showed binding and neutralizing activity (Fig 4) against the PRVABC59 strain of the Asian lineage (from the current outbreak). Therefore, these mouse scFvs may recognize different ZIKV epitopes with differential affinity that differ in accessibility or in their roles during infection. Indeed, the six scFvs demonstrated good ZIKV-binding capability and specificity (Figs 3D and 4A). scFv63-1 and scFv45-3 had the highest and second highest affinity, respectively, whereas scFv38-1 and scFv51-2 had moderate affinity and scFv5-1 and scFv7-2 antibody had the lowest affinity, reflecting that the panning was efficient in selecting a range of clones. These six clones didn’t show cross-reactivity with other flavivirus DENV2 E protein, suggesting specificity for ZIKV E protein. Zhao et al. [23] reported that five of the mAbs (ZV-2, ZV-48, ZV-54, ZV-64, and ZV-67) bound to recombinant ZIKV E DIII (domain III of ZIKV envelope protein) in a direct ELISA and were ZIKV-specific and in contrast, ZV-13 was cross-reactive to DENV1-4. Importantly, without *a priori* need of converting to full length IgG molecules, all six mice scFv antibodies neutralized live ZIKV (Fig 4B and 4C). A commercially available neutralizing anti-ZIKV antibody (ZKA64, Absolute Antibody, Oxford, UK, catalog # Ab00779-2.0) completely inhibited ZIKV MR766 infection in LLC-MK2 cells at the concentrations from 2 to 40 μg/ml, as demonstrated by Paul *et al*. [4]. This is in good agreement with our findings, as we observed that scFvs 45–3 and 63–1 completely inhibited ZIKV infectivity at concentrations between 20 to 50 μg/ml (Fig 4C). Our approach has the added advantage of generating Fc receptor-deficient antibodies, minimizing concern of antibody-dependent enhancement of infection.

ScFvs have been utilized against other infectious diseases. scFvs derived from vaccinated cynomolgus macaques were protective against the Ebola-related Marburg virus when tested in infected mice [24]. Similarly, a human scFv provided protection to mice infected with influenza virus [25]. scFvs are also promising candidates for anti-cancer therapeutics [26]. Therefore, scFvs are a potential therapeutic for ZIKV, due to their resistance to ADE, and could be used in diagnostics that can rapidly be generated as viruses evolve.

## Conclusions

The antibody ribosome display technology is a versatile platform that allowed the rapid (4 weeks) identification and isolation of six high-affinity scFvs to ZIKV E protein. These antibodies bind with affinities between 19 and 514 nM an apparent Kd as measured by ELISA. These scFvs recognized neutralization site(s) on the ZIKV E protein with differential antigen-binding affinities. The scFvs (45–3 and 63–1) with highest affinity could be used in therapeutic settings or diagnostics.

## Supporting information

S1 Fig Amino acid sequences of VH-Linker-VL of ZIKV E-specific mouse scFvs using Clustal Omega. FRs and CDRs are determined by the IMGT information system. Diversity was found predominantly in the CDR regions. A normal 20 amino acid linker [(G_4_S)_4_] joins the VH and VL chains. Alignments were colour coded according to residue property groups. AVFPMILW-red, DE-blue, RK-magenta, STYHCNGQ-green, others-grey.
(TIF)

S1 Table Nucleotide sequences of primers used. (DOCX)

S2 Table Analysis of VH and VL gene usage in the selected scFv clones. Nucleotide and amino acid differences in V-gene segment, excluding CDR3. The nucleotide sequences were analyzed using V-BASE2 (http://www.vbase2.org/vbscAb.php).
(DOCX)

S3 Table Plaque count from plaque reduction (PRNT) assay for ZIKVPRVABC59 neutralization by anti-ZIKV scFvs. (DOCX)
