# Supplementary figures and images for: Ribosome display for the rapid generation of high-affinity Zika-neutralizing single-chain antibodies
Source: PLoS One. 2018 Nov 16;13(11):e0205743. doi: 10.1371/journal.pone.0205743 (PMC6239285; doi:10.1371/journal.pone.0205743)

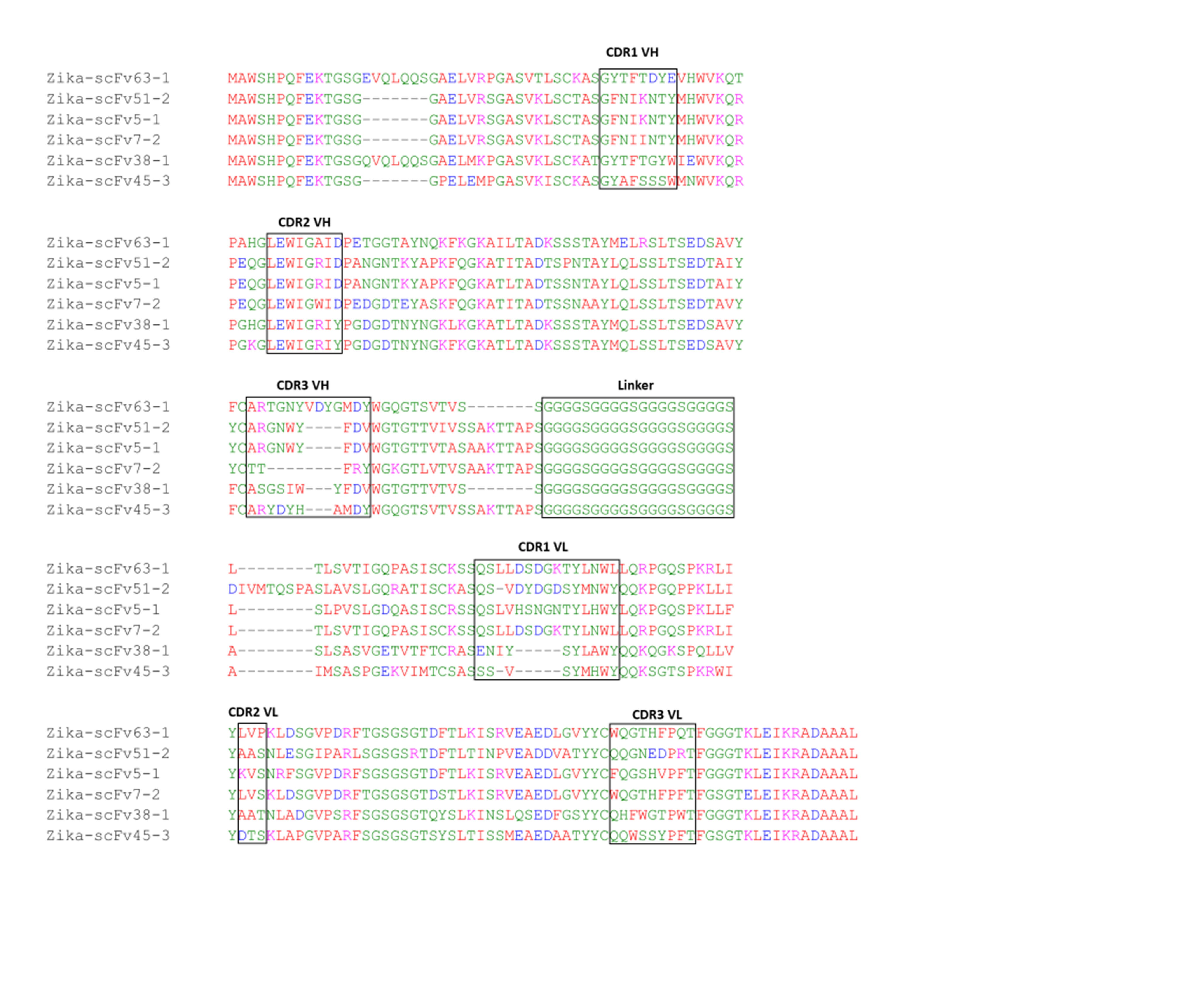

Supplement: S1 Fig — FRs and CDRs are determined by the IMGT information system. Diversity was found predominantly in the CDR regions. A normal 20 amino acid linker [(G4S)4] joins the VH and VL chains. Alignments were colour coded according to residue property groups. AVFPMILW-red, DE-blue, RK-magenta, STYHCNGQ-green, others-grey. (TIF) [file pone.0205743.s001.tif]
