# Supplementary material for: Ribosome display for the rapid generation of high-affinity Zika-neutralizing single-chain antibodies
Source: PLoS One. 2018 Nov 16;13(11):e0205743. doi: 10.1371/journal.pone.0205743 (PMC6239285; doi:10.1371/journal.pone.0205743)
Supplement: S1 Table — (DOCX) [file pone.0205743.s002.docx]

| **Primer name** | **Primer sequence (5’ – 3’)** |
| --- | --- |
|  | **VH primers** |
| MVH_F1 | CGAGAAGACCGGCAGCGGTGGGGCAGAGCTTGTGAAGCCA |
| MVH_F2 | CGAGAAGACCGGCAGCGGTGGAGGAGGCTTGATGCAACCT |
| MVH_F3 | CGAGAAGACCGGCAGCGGTGGACCTGAGCTGGAGATGCCT |
| MVH_F4 | CGAGAAGACCGGCAGCGGTGGACCTGGCCTGGTGAGACCT |
| MVH_F5 | CGAGAAGACCGGCAGCGGTGGGGGAGGCTTAGTGAAGCCT |
| MVH_F6 | CGAGAAGACCGGCAGCGGTGGGGCAGAGCTTGTGAAGCCA |
| AMVH_F7 | CGAGAAGACCGGCAGCGGTGGAGGGGGCTTGGTACAGCCT |
| MVH_F8 | CGAGAAGACCGGCAGCGGTGGGGCAGAGCTTGTGAGGTCA |
| MVH_F9 | CGAGAAGACCGGCAGCGGT GAKGTRMAGCTTCAGGAGTC |
| MVH_F10 | CGAGAAGACCGGCAGCGGT GAGGTBCAGCTBCAGCAGTC |
| MVH_F11 | CGAGAAGACCGGCAGCGGT CAGGTGCAGCTGAAGSASTC |
| MVH_F12 | CGAGAAGACCGGCAGCGGT GAGGTCCARCTGCAACARTC |
| MVH_F13 | CGAGAAGACCGGCAGCGGT CAGGTYCAGCTBCAGCARTC |
| MVH_F14 | CGAGAAGACCGGCAGCGGT CAGGTYCARCTGCAGCAGTC |
| MVH_F15 | CGAGAAGACCGGCAGCGGT CAGGTCCAGGTGAAGCAGTC |
| MVH_F16 | CGAGAAGACCGGCAGCGGT GAGGTGAASSTGGTGGAATC |
| MVH_F17 | CGAGAAGACCGGCAGCGGT GAVGTGAWGYTGGTGGAGTC |
| MVH_F18 | CGAGAAGACCGGCAGCGGT GAGGTGCAGSKGGTGGAGTC |
| MVH_F19 | CGAGAAGACCGGCAGCGGT GAKGTGCAMCTGGTGGAGTC |
| MVH_F20 | CGAGAAGACCGGCAGCGGT GAGGTGAAGCTGATGGARTC |
| MVH_F21 | CGAGAAGACCGGCAGC GGT GAGGTGCARCTTGTTGAGTC |
| MVH_F22 | CGAGAAGACCGGCAGCGGT GARGTRAAGCTTCTCGAGTC |
| MVH_F23 | CGAGAAGACCGGCAGCGGT GAAGTGAARSTTGAGGAGTC |
| MVH_F24 | CGAGAAGACCGGCAGCGGT CAGGTTACTCTRAAAGWGTSTG |
| MVH_F25 | CGAGAAGACCGGCAGCGGT CAGGTCCAACTVCAGCARCC |
| MVH_F26 | CGAGAAGACCGGCAGCGGT GATGTGAACTTGGAAGTGTC |
| MVH_F27 | C GAG AAG ACC GGC AGC GGT GAGGTGAAGGTCATCGAGTC |
|  |  |
| MVH_R1 | GGAGCCGCCGCCGCCGCCAGAACCACCACCACC***GGATCC***ACCACCACCCGAGGAAACGGTGACCGTGGT |
| MVH_R2 | GGAGCCGCCGCCGCCGCCAGAACCACCACCACC***GGATCC***ACCACCACCCGAGGAGACTGTGAGAGTGGT |
| MVH_R3 | GGAGCCGCCGCCGCCGCCAGAACCACCACCACC***GGATCC***ACCACCACCCGCAGAGACAGTGACCAGAGT |
| MVH_R4 | GGAGCCGCCGCCGCCGCCAGAACCACCACCACC***GGATCC***ACCACCACCCGAGGAGACGGTGACTGAGGT |
| MVH_R5 | GGAGCCGCCGCCGCCGCCAGAACCACCACCACC***GGATCC***ACCACCACCCGATGGGGCTGTTGTTTTGGC |
| MVH_R6 | GGAGCCGCCGCCGCCGCCAGAACCACCACCACC***GGATCC***ACCACCACCTGATGGGGGTGTTGTTTTGGC |
| MVH_R7 | GGAGCCGCCGCCGCCGCCAGAACCACCACCACC***GGATCC***ACCACCACCCGATGGGGCTGTTGTTTTGGC |
| MVH_R8 | GGAGCCGCCGCCGCCGCCAGAACCACCACCACC***GGATCC***ACCACCACCCGATGGGGCTGTTGTTTTGGC |
| MVH_R9 | GGAGCCGCCGCCGCCGCCAGAACCACCACCACC***GGATCC***ACCACCACCCGATGGGGCTGTTGTTTTGGC |
| MVH_R10 | GGAGCCGCCGCCGCCGCCAGAACCACCACCACC***GGATCC***ACCACCACCCGATGGGGCTGTTGTTTTGGC |
| MVH_R11 | GGAGCCGCCGCCGCCGCCAGAACCACCACCACC***GGATCC***ACCACCACCAGATGGGGGTGTCGTTTTGGC |
| MVH_R12 | GGAGCCGCCGCCGCCGCCAGAACCACCACCACC***GGATCC***ACCACCACCCGATGGGGCTGTTGTTTTGGC |
|  | **VL primers** |
| MVK_F1 | GGCGGCGGCGGCTCCGGTGGTGGT***GGATCC***GCAATCATGTCTGCATCTCC |
| MVK_F2 | GGCGGCGGCGGCTCCGGTGGTGGT***GGATCC***GCCTCCCTATCTGTATCTGTG |
| MVK_F3 | GGCGGCGGCGGCTCCGGTGGTGGT***GGATCC***GCCTCCCTATCTGCATCTGTG |
| MVK_F4 | GGCGGCGGCGGCTCCGGTGGTGGT***GGATCC***CTCACTTTGTCGGTTACCATT |
| MVK_F5 | GGCGGCGGCGGCTCCGGTGGTGGT***GGATCC***TCAGCCTCTTTCTCCCTGGGA |
| MVK_F6 | GGCGGCGGCGGCTCCGGTGGTGGT***GGATCC***TCCTCCCTGAGTGTGTCAGCA |
| MVK_F7 | GGCGGCGGCGGCTCCGGTGGTGGT***GGATCC***CTCTCCCTGCCTGTCAGTCTT |
| MVK_F8 | GGCGGCGGCGGCTCCGGTGGTGGT***GGATCC***CTCTCCCTGCCTGTCAGTCTT |
|  |  |
| MVKR | AGT**GCGGCCGC**ATCAGCCCGTTTTATTTCCAA |
|  |  |
|  | **Strep Tag II** |
| KzSTREPII | CGAATTCCACCATGGCCTGGAGCCATCCGCAGTTCGAGAAGACCGGCAGCGG |
|  | **T7 promoter** |
| RDT7 | CTATAGAAGG GTAATACGACTCACTATAGGGCGAATTCCACCATGGCC |
| T7 | TAATACGACTCACTATAGGG |
| SP6 | ATTTAGGTGACACTATAG |
