# Supplementary material for: Ribosome display for the rapid generation of high-affinity Zika-neutralizing single-chain antibodies
Source: PLoS One. 2018 Nov 16;13(11):e0205743. doi: 10.1371/journal.pone.0205743 (PMC6239285; doi:10.1371/journal.pone.0205743)
Supplement: S2 Table — Nucleotide and amino acid differences in V-gene segment, excluding CDR3. The nucleotide sequences were analyzed using V-BASE2 (http://www.vbase2.org/vbscAb.php). (DOCX) [file pone.0205743.s003.docx]

| **Clone** | **VH** | | | | | | **VL** | | | | |
| --- | --- | --- | --- | --- | --- | --- | --- | --- | --- | --- | --- |
|  | **V- seg alignment** | **D-seg**  **alignment** | **J- seg**  **alignment** | **Name** | **Differences**  **from germline**  **(nucleotides,**  **amino acids)^a^** | **VHCDR3** | **V- seg alignment** | **J- seg**  **alignment** | **Name** | **Differences**  **from germline**  **(nucleotides,**  **amino acids)^a^** | **VLCDR3** |
| 5-1 | V segment of the Igh-V15 VH15 family | DFL16.1, DFL16.3, DSP2.1 | JH1B6 mouse, JH1 mouse, JH2 mouse | musIGHV236 | (21, 10) | ARGNWYFDV | V segment of the IGKV1 subgroup | jk2, jk4, jk1 | musIGKV115 | (23, 11) | FQGSHVPFT |
| 7-2 | V segment of the Igh-VSM7 VH14 family | Not found | JH3 mouse, JH1B6 mouse, JH1 mouse | musIGHV247 | (26, 13) | TTFRY | V segment of the IGKV2 subgroup | Jk4, jk2, jk5 | musIGKV097 | (24, 9) | WQGTHFPFT |
| 38-1 | V segment of the Igh-VJ558 VH1 family | IGHD6-2*02, PseudoD2, IGHD2-14*01inv | JH1B6 mouse, JH1 mouse, JH2 mouse | musIGHV364 | (16, 12) | ASGSIWYFDV | V segment of the IGKV12/13 subgroup | jk1, jk2, jk4 | musIGKV170 | (22, 11) | QHFWGTPWT |
| 45-3 | V segment of the Igh-VJ558 VH1 family | DSP2.3, DSP2.4, DSP2.5 | JH4 mouse, JH2 mouse, JH1 mouse | musIGHV364 | (19, 9) | ARYDYHAMDY | V segment of the IGKV4/5 subgroup | Jk4, jk2, jk5 | musIGKV072 | (25, 10) | QQWSSYPFT |
| 51-2 | V segment of the Igh-V15 VH15 family | DFL16.1, DFL16.3, DSP2.1 | JH1B6 mouse, JH1 mouse, JH2 mouse | musIGHV236 | (21, 10) | ARGNWYFDV | V segment of the IGKV21 subgroup | jk1, jk2, jk4 | musIGKV208 | (15, 8) | QQGNEDPRT |
| 63-1 | V segment of the Igh-VJ558 VH1 family | DSP2.1, DSP2.5, DSP2.7 | JH4 mouse, JH2 mouse, JH1 mouse | musIGHV287 | (9, 4) | ARTGNYVDYGMDY | V segment of the IGKV2 subgroup | jk1, jk2, jk4 | musIGKV097 | (22, 9) | WQGTHFPQT |
