# Supplementary material for: Ribosome display for the rapid generation of high-affinity Zika-neutralizing single-chain antibodies
Source: PLoS One. 2018 Nov 16;13(11):e0205743. doi: 10.1371/journal.pone.0205743 (PMC6239285; doi:10.1371/journal.pone.0205743)
Supplement: S3 Table — (DOCX) [file pone.0205743.s004.docx]

|  | **Plaque count at each point** | | | | |
| --- | --- | --- | --- | --- | --- |
| **ScFv concentration (µg/ml)** | 1 | 10 | 20 | 50 | 100 |
| **First set** |  |  |  |  |  |
| scFv5-1 | 43 | 34 | 33 | 23 | 21 |
| scFv7-2 | 39 | 28 | 20 | 19 | 10 |
| scFv38-1 | 56 | 55 | 39 | 17 | 11 |
| scFv45-3 | 47 | 46 | 40 | 10 | 5 |
| scFv51-2 | 45 | 43 | 32 | 20 | 9 |
| scFv63-1 | 40 | 23 | 10 | 2 | 3 |
| Ebov4-2 (Negative control) | 66 | 57 | 63 | 65 | 64 |
| Virus control | 62 | 65 | 67 | 60 | 65 |
| **Second set** |  |  |  |  |  |
| scFv5-1 | N.D | N.D | N.D | N.D | N.D |
| scFv7-2 | 41 | 33 | 18 | 8 | 4 |
| scFv38-1 | 49 | 35 | 31 | 25 | 24 |
| scFv45-3 | 30 | 23 | 15 | 11 | 10 |
| scFv51-2 | 38 | 31 | 15 | 8 | 6 |
| scFv63-1 | 36 | 22 | 17 | 11 | 9 |
| Ebov4-2 (Negative control) | 51 | 51 | 53 | 50 | 52 |
| Virus control | 51 | 55 | 49 | 49 | 49 |
| **Third set** |  |  |  |  |  |
| scFv5-1 | 22 | 18 | 17 | 13 | 11 |
| scFv7-2 | 19 | 18 | 10 | 11 | 5 |
| scFv38-1 | 22 | 18 | 10 | 13 | 6 |
| scFv45-3 | 22 | 13 | 9 | 6 | 2 |
| scFv51-2 | 18 | 15 | 10 | 8 | 5 |
| scFv63-1 | 19 | 15 | 10 | 6 | 1 |
| Ebov4-2 (Negative control) | 34 | 33 | 35 | 31 | 36 |
| Virus control | 32 | 35 | 34 | 33 | 34 |

N.D: Not determined
